# The Homolog of the Five SH3-Domain Protein (HOFI/SH3PXD2B) Regulates Lamellipodia Formation and Cell Spreading

**DOI:** 10.1371/journal.pone.0023653

**Published:** 2011-08-23

**Authors:** Árpád Lányi, Mónika Baráth, Zalán Péterfi, Gábor Bőgel, Anna Orient, Tünde Simon, Enikő Petrovszki, Katalin Kis-Tóth, Gábor Sirokmány, Éva Rajnavölgyi, Cox Terhorst, László Buday, Miklós Geiszt

**Affiliations:** 1 Department of Immunology, University of Debrecen Medical and Health Science Center, Debrecen, Hungary; 2 Research Centre for Molecular Medicine, University of Debrecen Medical and Health Science Center, Debrecen, Hungary; 3 Department of Physiology, Faculty of Medicine, Semmelweis University, Budapest, Hungary; 4 Department of Medical Chemistry, Molecular Biology and Pathobiochemistry, Faculty of Medicine, Semmelweis University, Budapest, Hungary; 5 Division of Immunology, Beth Israel Deaconess Medical Center, Harvard Medical School, Boston, Massachusetts, United States of America; 6 Institute of Enzymology, Biological Research Center, Hungarian Academy of Sciences, Budapest, Hungary; 7 Peroxidase Enzyme Research Group, Semmelweis University and the Hungarian Academy of Sciences, Budapest, Hungary; Semmelweis University, Hungary

## Abstract

Motility of normal and transformed cells within and across tissues requires specialized subcellular structures, e.g. membrane ruffles, lamellipodia and podosomes, which are generated by dynamic rearrangements of the actin cytoskeleton. Because the formation of these sub-cellular structures is complex and relatively poorly understood, we evaluated the role of the adapter protein SH3PXD2B [HOFI, fad49, Tks4], which plays a role in the development of the eye, skeleton and adipose tissue. Surprisingly, we find that SH3PXD2B is requisite for the development of EGF-induced membrane ruffles and lamellipodia, as well as for efficient cellular attachment and spreading of HeLa cells. Furthermore, SH3PXD2B is present in a complex with the non-receptor protein tyrosine kinase Src, phosphorylated by Src, which is consistent with SH3PXD2B accumulating in Src-induced podosomes. Furthermore, SH3PXD2B closely follows the subcellular relocalization of cortactin to Src-induced podosomes, EGF-induced membrane ruffles and lamellipodia. Because SH3PXD2B also forms a complex with the C-terminal region of cortactin, we propose that SH3PXD2B is a scaffold protein that plays a key role in regulating the actin cytoskeleton via Src and cortactin.

## Introduction

Molecular scaffolds are crossroads where parallel signal transduction pathways of vital cellular functions including cellular attachment or migration are integrated [1], [2], [3]. Scaffold proteins usually consist of multiple domains that can serve independently or cooperatively as docking sites to recruit the appropriate set of proteins in a temporally and spatially ordered fashion. The Pleckstrin homology (PH) and Phox homology (PX) domains bind different phosphoinositides and are believed to be responsible for anchoring adaptor proteins to various microdomains of the cell membrane [4]. PX-domains are frequently found in combination with Src-homology3 (SH3) domains that bind to proline-rich sequences. Although the molecular details of interaction between PX-domain and phospoinositides are well-mapped, the complexity of the *in vivo* functions of this PX/SH3 protein module is not well understood.

Recently, a distinct member of PX/SH3 protein family, FISH/Tks5, comprised of FIve SH3 domains and an N-terminal PX-domain [5], has emerged as a central player of the protein scaffold regulating podosome formation in Src-transformed fibroblast cells [6]. FISH appears to be requisite for the organization of podosomes [7], actin-rich structures which are implicated in the invasion of tumor cells as well as in the migration of macrophages or dendritic cells within tissues [8].

In addition to podosomes, there are other organelles that control motility and invasion. Lamellipodia and filopodia form at the leading edge of the migrating cell generated by actin polymerization at the barbed end of the growing filaments [9], [10]. Lamellipodia are shallow protruding structures comprised of a dense array of branched actin filaments, while filopodia are rod-like protrusions built from a bundle of actin filaments [11], [12], [13]. Lamellipodia and podosomes are enriched in cortactin [14], [15], a scaffold protein that is a substrate for Src [15]. Cortactin has been shown to enhance cell migration in transwell systems, as well as in wound healing assays [16], [17], [18], [19]. Moreover, migration, generation of persistent lamellipodia, invadopodia and efficient cell spreading can be inhibited by siRNAs targeting cortactin [20], [21]. However, experiments with cortactin null murine fibroblasts showed an impairment only in growth factor induced actin cytoskeleton reorganization and migration, but not in generation of lamellipodia[22]. Nevertheless, overexpression of cortactin in multiple types of human tumors e.g. in gastric, liver and breast cancer suggests that cortactin is a key regulator of these processes *in vivo* [23], [24], [25].

Recently several groups including ours have identified a homologue of FISH/Tks5 referred to as HOFI/Tks4/fad49. Subsequently, another name, SH3PXD2B was also assigned to the protein that for simplicity will be used in this paper. In *in vitro* and *ex vivo* studies the murine ortologue of SH3PXD2B was shown to play an important role in the formation of functional podosomes [26], production of reactive oxygen species (ROS) by tumor cells [27], [28] and in the differentiation of white adipose tissue [29]. In two independent mice models, the absence of SH3PXD2B was found to profoundly impair normal development causing runted growth, craniofacial and skeletal abnormalities, hearing impairment, glaucoma and the virtual absence of white adipose tissue [30], [31]. In humans, SH3PXD2B-deficiency is responsible for the development of Frank-Ter Haar syndrome (MIM 249420) [30]. Despite its impact on development and cell physiology, the molecular machinery operated by SH3PXD2B is poorly understood.

In the present study we characterize the human orthologue of SH3PXD2B and examine potential mechanisms and functions that are controlled by SH3PXD2B. We show that SH3PXD2B, upon activation by growth factors or expression of constitutively active Src, associates with dynamic assemblies of the actin cytoskeleton *e.g.* lamellipodia and podosomes, independent of lipid binding via its PX-domain. Furthermore, we found SH3PXD2B in a complex with two central regulators of the actin cytoskeleton; Src and cortactin. The latter interaction required the C-terminal part and an intact SH3-domain of cortactin. When SH3PXD2B was eliminated by RNA interference we detected a marked impairment in growth factor-induced membrane ruffling and in the generation of lamellipodia and hence, as our experiments show, SH3PXD2B plays an important role in cellular attachment and cell spreading.

## Results

### Expression of SH3PXD2B in tumor-derived cell lines and primary cells

**Figure 1:**
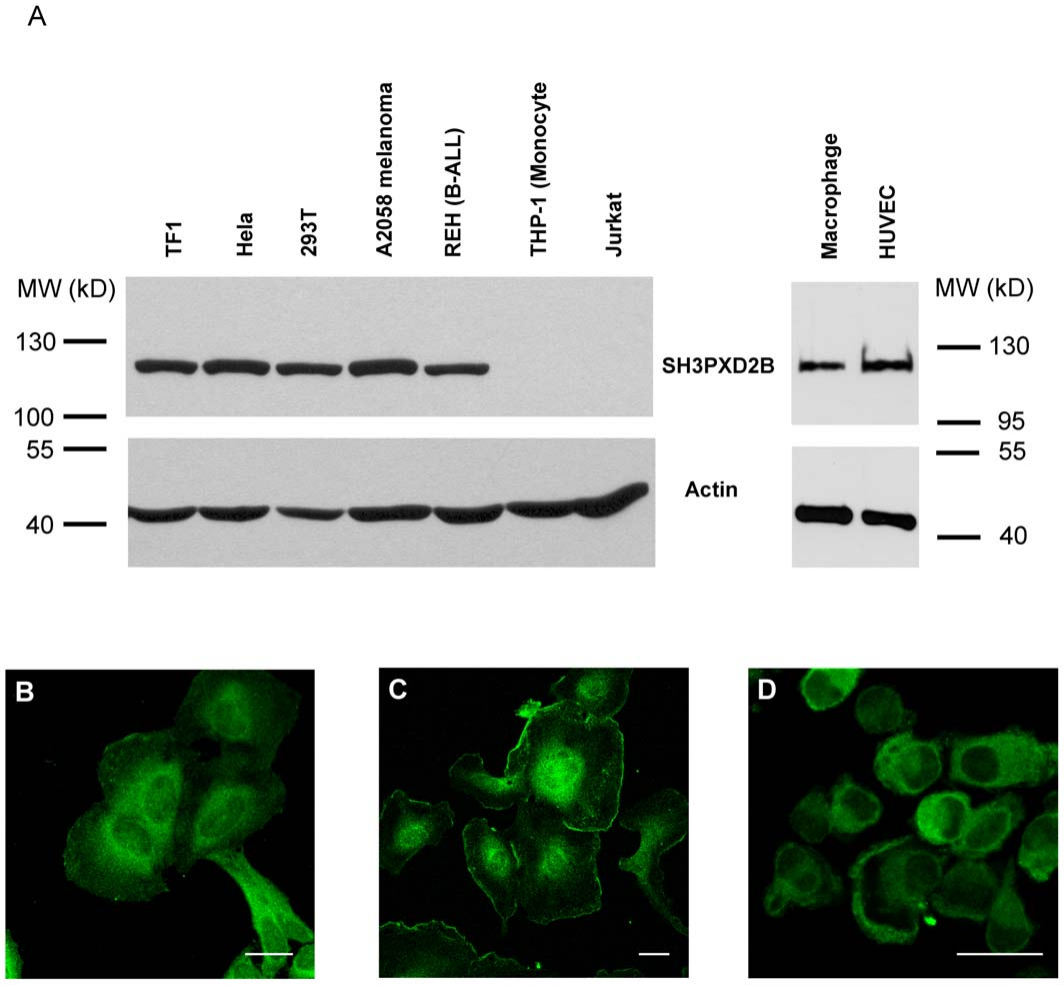
Expression of SH3PXD2B in various human cell lines and primary cells. (**A**) Expression of the SH3PXD2B protein in various transformed cell lines (left panel), macrophages and HUVEC (right panel). SH3PXD2B was detected by the affinity purified antibody described in Materials and Methods. Actin was used as a loading control. (**B–D**) Intracellular localization of SH3PXD2B. SH3PXD2B protein in HeLa cells (**B**), in HUVECs (**C**) and in primary human macrophages (**D**) was visualized by immunofluorescence. Cells were grown on coverslips and processed as detailed in Materials and Methods. Bars indicate 20 µm.

Because FISH/Tks5 appeared implicated in malignancies, we assessed SH3PXD2B expression in tumor-derived cell lines. To this end we cloned the full-length cDNA encoding human SH3PXD2B (Genbank, DQ109556, AAZ99795) and raised a polyclonal antibody directed against the N-terminal region of the protein. This antibody, which recognized the predicted protein of approximately 120 kD in a western blotting experiment (**Figure 1A**), detected high levels of SH3PXD2B in several human tumor cell lines *e.g.* in HeLa, 293T, the acute B-cell leukemia line REH, the erythroleukemia cell line TF1 and the A2058 melanoma. (**Figure 1A** left panel). As it was not found in Jurkat T cells or in the acute monocytic leukemia cell line THP1, SH3PXD2B expression appears not essential to maintain continuous cell proliferation. Importantly, SH3PXD2B expression is not restricted to tumor-derived cell lines, as primary cells including human macrophages and human umbilical vein endothelial cells (HUVEC) also expressed the protein (**Figure 1A** right panel).

Next, we examined the intracellular localization of SH3PXD2B in the tumor-derived HeLa cells or in primary cells, e.g. HUVEC and monocyte-derived macrophages. SH3PXD2B was present in or adjacent to the plasma membrane and in the perinuclear region of both transformed and primary cells (**Figure 1B–D**). Although perinuclear staining was prominent in HeLa cells and in HUVEC, SH3PXD2B did not co-localize with either the endosomal marker transferrin receptor or the Endoplasmic Reticulum(ER)-specific protein disulfide-isomerase (data not shown).

### In membrane ruffles, lamellipodia and podosomes SH3PXD2B co-localizes with cortactin

**Figure 2:**
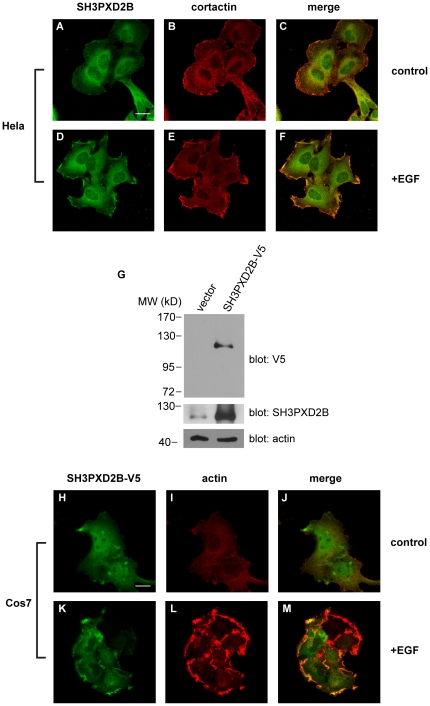
Epidermal growth factor receptor activation induces the co-localization of SH3PXD2B with cortactin and F-actin in membrane ruffles and lamellipodia. Serum starved cells were left untreated (**A–C** and **H–J**) or treated with 100 ng/ml EGF (**D–F & K–M**) for 10 minutes. Cells were then fixed and processed for immunofluorescence. Endogenous expression of SH3PXD2B and cortactin in HeLa cells (**A–F**) were detected using SH3PXD2B-specific rabbit polyclonal or cortactin-specific mouse monoclonal antibodies. Cellular localization of SH3PXD2B in Cos7 cells transfected with a construct encoding an SH3PXD2B fusion protein carrying a C-terminal V5 epitope tag (**H–M**) was visualized using a mouse anti-V5 monoclonal antibody and anti-mouse Alexa488 (green). Filamentous actin (F-actin) was stained with Alexa568-labeled phalloidin. Bars indicate 20 µm. Efficiency of SH3PXD2B-V5 overexpression was analyzed by western blotting (G).

Because FISH/Tks5 and SH3PXD2B were required for the generation of Src-induced podosomes [7], [26], [32], [33] we reasoned that SH3PXD2B may also play a role in the generation of other dynamic membrane structures. To test this, we induced the formation of membrane ruffles and lamellipodia in serum starved HeLa cells with epidermal growth factor (EGF) (**Figure 2A–F**). Under resting conditions SH3PXD2B localized to the cytosol often in the perinuclear region, whilst staining of the plasma membrane appeared to be minimal (**Figure 2A**). EGF-induced membrane ruffles and lamellipodia, however, were enriched in SH3PXD2B (**Figure 2D**). When cortactin, a marker for cortical actin polymerization was visualized with a specific antibody, we observed intense cortactin accumulation in the EGF-induced membrane protrusions where cortactin co-localized with SH3PXD2B (**Figure 2E & 2F**). Co-localization of SH3PXD2B with cortactin was evident in 100% of cells that developed membrane ruffles or lamellipodia in response to EGF stimulation.

**Figure 3:**
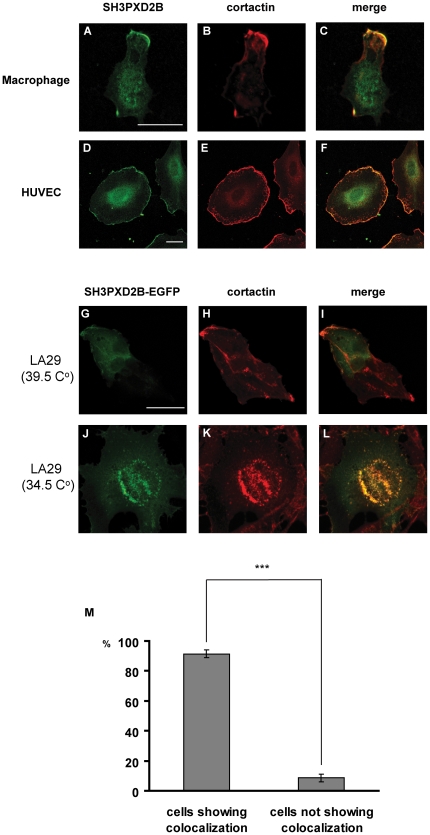
Co-localization of SH3PXD2B with cortactin in HUVEC, macrophages and LA29ts fibroblasts. Human monocyte-derived macrophages (**A–C**), HUVEC (**D–F**) were stained as described for Figure 2A–F. (**G–L**) Rat-1 LA29 fibroblast cells carrying a temperature sensitive mutant form of Src were grown on coverslips and transfected with an SH3PXD2B-EGFP construct. Cultures were kept at the nonpermissive temperature (39.5°C) for 24 hours. Next, half of the cells were grown further at the nonpermissive temperature until the end of the experiment (**G–I**). The rest of the cells were cultured at the permissive temperature (34.5°C) overnight (**J–L**). Coverslips were prepared for immunfuorescence as described in Materials and Methods. Cortactin and SH3PXD2B were visualized as described in the legend for Figure 2. Bars represent 20 µm. Co-localization between SH3PXD2B and cortactin was observed in over 90% of the cells grown at the permissive temperature (M). Over 100 cells were evaluated for each independent experiment. Error bars represent the Standard Error of the Mean, SEM. ***: p<0.001, n = 3.

To corroborate co-localization of SH3PXD2B with components of the actin cytoskeleton, we examined its potential co-localization with actin filaments in EGF-treated Cos-7 cells. For this set of experiments, SH3PXD2B was tagged with a C-terminal V5 epitope and the construct encoding the fusion protein was transfected into Cos-7 cells. Western blots of cell extracts derived from the transfected cells contained a single band reacting with the V5-specific antibody. The molecular weight of the expressed V5-fusion protein was found to be identical to that of SH3PXD2B (**Figure 2G** **top panel**) indicating that the protein was full-length and unprocessed. To assess the extent of over-expression the same blots were stripped and re-probed with an SH3PXD2B-specific antibody (**Figure 2G** **middle panel**) which labeled the same band in addition to the less prominent, endogenously expressed SH3PXD2B. Similar to endogenously expressed SH3PXD2B, the V5-epitope tagged fusion protein localized to membrane ruffles where it co-localized with polymerized actin (**Figure 2K–M**). Co-localization of V5-SH3PXD2B with actin was observed in 100% of cells that developed membrane ruffles or lamellipodia. Co-localization of endogenous SH3PXD2B with cortactin was also evident in primary cells e. g. in lamellipodia at the leading edge formed by polarized, migrating monocyte-derived macrophages (**Figure 3A–C**) or HUVECs (**Figure 3D–F**).

Because cortactin is one of the podosome markers, we also tested its co-localization with SH3PXD2B in Src-induced podosomes. To this end, we used a modified Rat-1 cell line, LA29ts, that carries a temperature sensitive mutant form of Src [34], [35], [36]. In LA29ts cells, at the nonpermissive temperature (39.5°C), Src is in an inactive conformation which is rapidly converted to the active conformation when cells are cultured at the permissive temperature (34.5°C). As predicted, activation of Src induces the appearance of podosomes (**Figure 3J–L** **.**). Importantly, SH3PXD2B primarily localized to the cytosol in LA29ts cells transfected with the full-length SH3PXD2B-EGFP construct when they were kept at the nonpermissive temperature, (**Figure 3G**). By contrast, when cells were grown at the permissive temperature, as expected, SH3PXD2B localized to the forming rosettes of podosomes (**Figure 3J**). To visualize podosomes and other membrane structures, slides were counterstained with a cortactin-specific antibody. Merging of the confocal images revealed co-localization of SH3PXD2B and cortactin in Src-induced podosomes (**Figure 3L**), which was not observed in the absence of active Src at the nonpermissive temperature (**Figure 3I**). Co-localization between SH3PXD2B and cortactin at the permissive temperature was evident in over 95% of the cells (**Figure 3M**). Functional activity of cortactin- and SH3PXD2B-rich structures visually identified as podosomes were further analyzed in standard gelatin-degradation assays. As shown in Figure S1, gelatinase activity was readily detectable at the permissive temperature suggesting that the developing podosomes were functionally active.

Taken together, SH3PXD2B closely follows the pattern of cortactin's intracellular movement into multiple actin-based membrane structures including EGF-induced membrane ruffles and lamellipodia or Src-induced podosomes, suggesting that SH3PXD2B together with cortactin may function as a general regulator of the actin cytoskeleton in various mammalian cells.

### SH3PXD2B associates with cortactin and Src

**Figure 4:**
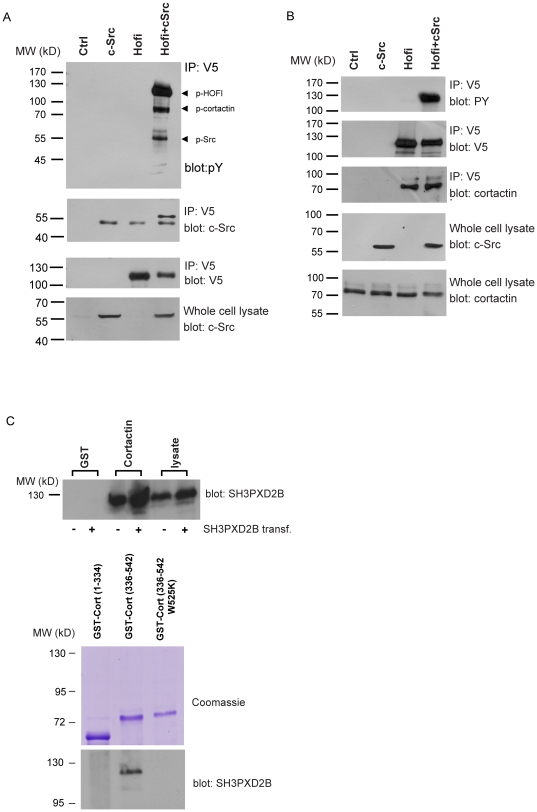
SH3PXD2B associates with Src and cortactin. (**A & B**) 293T cells were transfected with constructs expressing full-length SH3PXD2B carrying a C-terminal V5 epitope tag, Src or both. 48 hours past transfection cells were lysed and SH3PXD2B was precipitated using a V5-specific antibody. Immunoprecipitated proteins were blotted onto nitrocellulose membranes and detected with the indicated specific antibodies. The 52 kD-bands present in panel A. correspond to the heavy chain of the antibody used to precipitateV5-tagged SH3PXD2B. (**C**): In GST pull-down experiments recombinant GST or a GST-cortactin fusion protein were precipitated with glutathion-agarose beads from 293T cell extracts transfected (+) or not (−) with SH3PXD2B (top panel). In similar pull-down experiments GST- fusion proteins representing the N-terminal 334 amino acid region (Cort (1–334)) or the C-terminal region (Cort (336-542)) or its substituent (Cort (336–542(W525K)) were precipitated from lysates of 293T cells (bottom panel). SH3PXD2B was detected by western blotting. GST-proteins (30% of the amounts used in precipitation reactions) are visualized by Coomassie staining (middle panel) Cort: cortactin.

Several key regulators of the actin cytoskeleton including SH3PXD2B, cortactin, paxillin are phosphorylated by Src. To gain further insight into SH3PXD2B function we used co-immuno-precipitation to identify SH3PXD2B-associated phosphoproteins that are phosphorylated by Src [14], [15], [26], [37]. 293T cells were transiently transfected with the construct encoding a C-terminal V5-tagged full-length SH3PXD2B protein alone or in combination with a construct expressing Src (**Figure 4A**). Transfected cells were lysed 48 hours after transfection and V5-SH3PXD2B was immunoprecipitated with a V5-specific antibody. Western blotting with a phosphotyrosine-specific antibody revealed three dominant bands with molecular weights of 120 kD, 80 kD and 60 kD (**Figure 4A** **top panel**). Phosphorylation requires at least a transient interaction between the kinase and its substrate; thus, we tested whether SH3PXD2B and Src form a complex. We found that Src was readily precipitated from cell extracts prepared from cells co-transfected with V5-SH3PXD2B and Src **(** **Figure 4A** **panel Src)**. Src was absent in immunoprecipitates obtained from samples transfected with Src or V5-SH3PXD2B alone. The 120 kD and the 60 kD proteins in **Figure 4A** were SH3PXD2B and Src, respectively, as judged by stripping and re-probing the anti-pY blots with the corresponding specific antibodies (not shown).

The rapid cellular redistribution of SH3PXD2B in Src-transformed Rat-1 cells, together with its co-localization with cortactin in multiple cell types prompted us to examine potential association of SH3PXD2B with cortactin. In the above described experimental system V5 epitope tagged SH3PXD2B protein co-precipitated cortactin from lysates of 293T cells (**Figure 4B**). Furthermore, 80 kD phosphoprotein in **Figure 4A** was identified as phosphorylated cortactin by re-probing the phospho-protein blots with an anti-cortactin antibody (not shown). Although phosphorylated forms of both SH3PXD2B and cortactin were present in the complex (**Figure 4A**), the SH3PXD2B/cortactin complex was also found without Src transfection suggesting that complex formation may be independent of tyrosine phosphorylation (**Figure 4B**). In the converse experiments we used GST-tagged cortactin to precipitate SH3PXD2B from lysates of 293T cells in which SH3PXD2B was transfected or endogenously expressed. As shown in **Figure 4C** (**top panel**), SH3PXD2B was precipitated by the GST-cortactin from cell extracts containing endogenous or elevated levels of SH3PXD2B. In further pull-down experiments we localized the site of interaction to the C-terminal of cortactin corresponding to amino acids 336–542 **(** **Figure 4C** **middle and bottom panels)**. The interacting C-terminal region includes the Calpain cleavage site, the proline-rich region with numerous tyrosine and serine phosphorylation sites and the SH3-domain of cortactin, partaking in protein interacions with chief regulators of the cytoskeleton [20], [21], [38]. Because the SH3-domain of cortactin seemed to be good candidate for SH3PXD2B binding we assessed the binding of the C-terminal-cortactin-GST fusion construct with the W525K substitution. Changing the conserved tryptophan residue to lysine is known to abolish binding to canonical SH3-binding sites in various SH3-domains[39], [40]. As shown in **Figure 4C** (bottom panel), the GST-cortactin[336–542(W525K)] protein failed to precipitate SH3PXD2B suggesting that their interaction required an intact SH3-domain on cortactin.

Taken together, we identified Src and cortactin as parts of complexes containing SH3PXD2B and we presented data suggesting that cortactin binds to an SH3PXD2B-containing complex through its SH3 domain.

### The PX-domain SH3PXD2B binds PtdIns lipids

**Figure 5:**
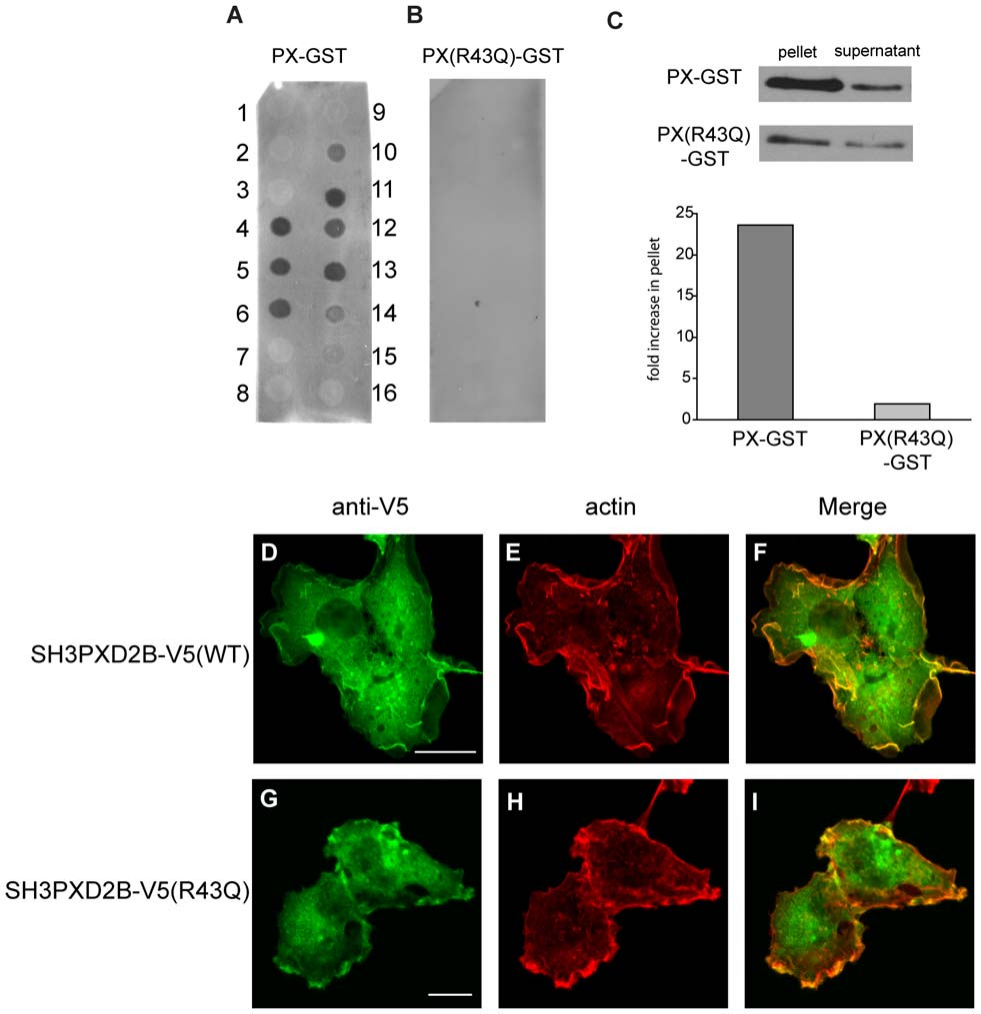
Characterization of the lipid-bindig domain of SH3PXD2B. (**A and B**) The lipid specificity of the PX-domain of SH3PXD2B was tested in protein-lipid overlay assay experiments using a recombinant GST fusion protein that contained the first 130 amino acid region of SH3PXD2B (**panel A**, PX-GST) or its derivative containing the R43Q substitution within the PX-domain (panel **B**, PX(R43Q). Layout of the PIP strip was the following: **1**. Lysophosphatidic acid **2**. Lyso-phosphatidylcholine **3**. Phosphatidylinositol (PtdIns) **4**. PtdIns(3)P **5**. PtdIns(4)P **6**. PtdIns(5)P **7**. Phosphatidylethanolamine **8**. Phosphatidylcholine **9**. Sphingosine 1-phosphate **10**. PtdIns (3,4)P_2_ **11**. PtdIns(3,5)P_2_ **12**. PtdIns(4,5)P_2_ **13**. PtdIns(3,4,5)P_3_ **14**. Phosphatidic acid **15**. Phosphatidylserine **16**. Blank. (**C**) Liposomes containing Phosphatidylcholine and PtdIns(3)P were mixed with recombinant PX-GST or PX(R43Q)-GST and centrifuged at at 80000 g for 20 minutes. Pellet and supernatant were separated, boiled and then subjected to SDS- polyacrylamide gel electrophoresis and western blotting as described in Materials and Methods. Blots were developed with a GST-specific antibody (top panels). Distribution of the PX-GST proteins between the liposome (pellet) and the aqueous phase (supernatant) was determined by densitometry (bottom panel). (**D–I**) Constructs expressing wild-type SH3PXD2B-V5 [SH3PXD2B-V5(WT)] or the SH3PXD2B-V5 with the R43Q amino acid substitution [SH3PXD2B-V5(R43Q)] were transfected into COS-7 cells (Panels **D–F** and **G–I**, respectively). 24 hours past transfection cells were serum depleted and activated with EGF as described for **Figure 2**. Actin and SH3PXD2B-V5 were visualized as described in the legends for Figure 2H–M. Bars represent 20 µm.

Binding of the PX-domain to specific membrane lipids is believed to contribute to the appropriate cellular localization of PX-SH3-domain proteins [41]. Hence, we examined the specificity of the PX-domain of SH3PXD2B towards various PtdIns lipids in a protein-lipid overlay assay. As shown in **Figure 5A**, a bacterially expressed GST-PX-domain fusion protein specifically bound to phosphorylated forms of phosphatidylinositol (PtdIns) however, interaction with phosphatidic acid (PA) was also detected. These binding characteristics were reminiscent to the PX-domains of FISH/Tks5 and p47^phox^ [32], [42], [43]. In addition to binding all three PtdIns-monophosphates, -diphosphates, binding to PtdIns (3,4,5)P_3_ triphosphate was readily detected. Interestingly, interaction with PtdIns(3,4)P_2_ was rather weak compared to that of PtdIns(3,5)P_2_ (**Figure 5A**). This finding is in line with those by Hishida et al. [29], showing strong binding of PtdIns(3,5)P_2_ but not PtdIns(3,4)P_2_ to the PX-domain of murine SH3PXD2B. Next we changed the Arginine residue at position 43 to Glutamine (R43Q). Changing the homologous Arginine residue in p47^phox^ and FISH/Tks5 eliminated binding to both PtdIns(3)P and PA [32], [42], [43]. As shown in **Figure 5B** the R43Q substitution abolished binding of the PX-domain to immobilized membrane lipids. To further corroborate these findings we generated liposomes of Phosphatidylcholine and PtdIns(3)P and incubated them with the GST-fusion protein containing the wild-type or the R43Q mutant SH3PXD2B PX-domain. As shown in **Figure 5C**, the wild-type PX-domain is predominantly found in the liposome fraction (pellet), while the R43Q mutant was only slightly enriched in the pellet.

Abram *et al.* suggested that the PX-domain is necessary and sufficient to drive podosomal localization of FISH/Tks5 in Src-transformed NIH3T3 cells [32]. Furthermore, Oikawa et al. demonstrated the importance of binding to PtdIns(3,4)P_2_ lipids by the PX-domain in this process [6]. We reasoned that a functional PX-domain should be essential for the proper re-localization of SH3PXD2B into membrane ruffles in EGF-stimulated cells. We introduced the R43Q substitution into the full-length protein and compared the cellular localization of the transfected full-length SH3PXD2B-V5 and the SH3PXD2B-V5(R43Q) fusion proteins in response to EGF stimulation. Interestingly, similar to its wild-type counterpart, the R43Q mutant protein localized to EGF-induced membrane ruffles, suggesting that a functional PX-domain is not essential for correct cellular localization of SH3PXD2B. **(**compare **Figure 5D & 5G** **with 2H)**. Furthermore, co-localization of SH3PXD2B with polymerized actin was equally prominent **(**compare **Figure 5F & 5I** **)**. Together these experiments suggested that phospholipid binding of the SH3PXD2B PX-domain does not play a major role in directing SH3PXD2B to lamellipodia or membrane ruffles.

### Development of lamellipodia and efficient cell spreading is impaired in the absence of SH3PXD2B

Because SH3PXD2B co-localized with cortactin in EGF-induced membrane ruffles and lamellipodia, we examined whether SH3PXD2B plays an active role in the organization of these structures.

**Figure 6:**
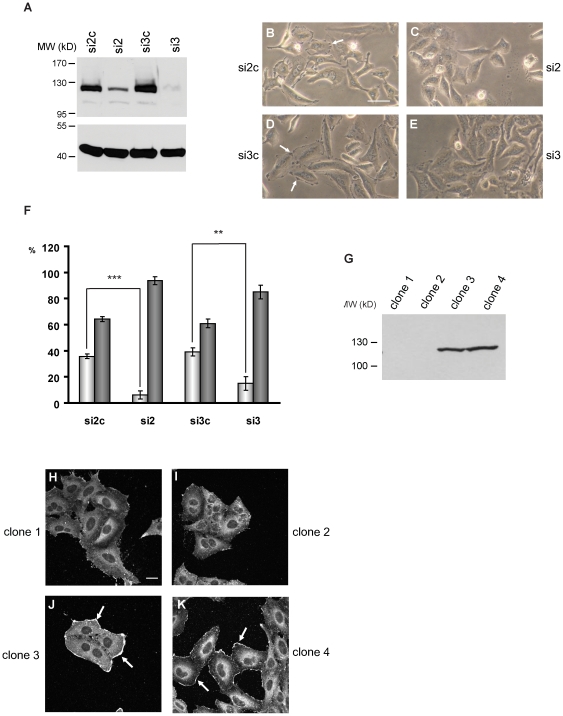
SH3PXD2B is essential for generation of membrane ruffles and lamellipodia. HeLa cells were grown on coverslips at subconfluent cell densities. Cells were transfected with double stranded control siRNA oligonucleotides (si2c and si3c) or siRNAs specific for SH3PXD2B (si2 and si3). (**A**) SH3PXD2B levels in cell lysates on the day of the functional assays were determined by western blot analysis. (**B–E**) At day 2 serum-starved cells were stimulated with EGF, fixed and visualized by phase contrast microscopy. White arrows show membrane ruffles and lamellipodia. (**F**) EGF-induced ruffling in cells transfected with the indicated siRNA-s were quantified at 10 minutes following activation. Percentages of cells with (columns shaded light grey) or without the characteristic membrane ruffling (columns shaded dark grey) are depicted. 100 cells were evaluated for each independent experiment (n = 4). Error bars represent the Standard Error of the Mean, SEM. ***: p<0.001, **: p<0.005. (G) Expression levels of SH3PXD2B in independent HeLa clones stably expressing SH3PXD2B-specific shRNA (clone 1 and clone 2.) or in two controls (clone 3 and clone 4). EGF-stimulated HeLa cell lines with diminished SH3PXD2B expression (**H & I**) or with normal SH3PXD2B expression (**J & K**) were stained for cortactin as described above. White arrows indicate lamellipodia rich in cortactin. Bars represent 20 µm.

To test this hypothesis, SH3PXD2B expression in HeLa cells was silenced by transient transfection of specific siRNAs (si2, si3) targeting different regions of the transcript. The efficiency of gene silencing exceeded 80% in all experiments typically approaching 90–95% (**Figure 6A**). Stimulation of HeLa cells with EGF resulted in rapid development of membrane ruffles in cells transfected with control siRNA (shown by white arrows in **Figure 6B & 6D**). By contrast, in cells transfected with SH3PXD2B-specific siRNA formation of membrane ruffles and lamellipodia was strongly reduced (**Figure 6C & E**). The frequency of membrane ruffling was determined by evaluating a minimum of 100 cells for each cell population in four independent experiments (**Figure 6F**).

In a second set of experiments we created stable HeLa clones expressing SH3PXD2B-specific small heteroduplex RNAs (shRNA) or control shRNA (with target sequences identical with si3 and si3c) from a vector carrying a puromycin resistance cassette. After antibiotic selection cell lines with no SH3PXD2B expression (Clones 1 and 2) and with normal SH3PXD2B expression (Clones 3 and 4) were studied further (**Figure 6G**). When stimulated with EGF, cortactin translocated rapidly into membrane ruffles and lamellipodia in control cells (indicated by white arrows in **Figure 6J & 6K**), while translocation of cortactin in cells with diminished SH3PXD2B content was less prominent (**Figure 6H & 6I**). Furthermore, we observed that 90–95% of the cells with normal SH3PXD2B expression generated lamellipodia exceeding 10 µm (**Figure 6J & 6K**) while such lamellipodia in cells with decreased SH3PXD2B content was only sporadically observed (**Figure 6H & 6I**).

**Figure 7:**
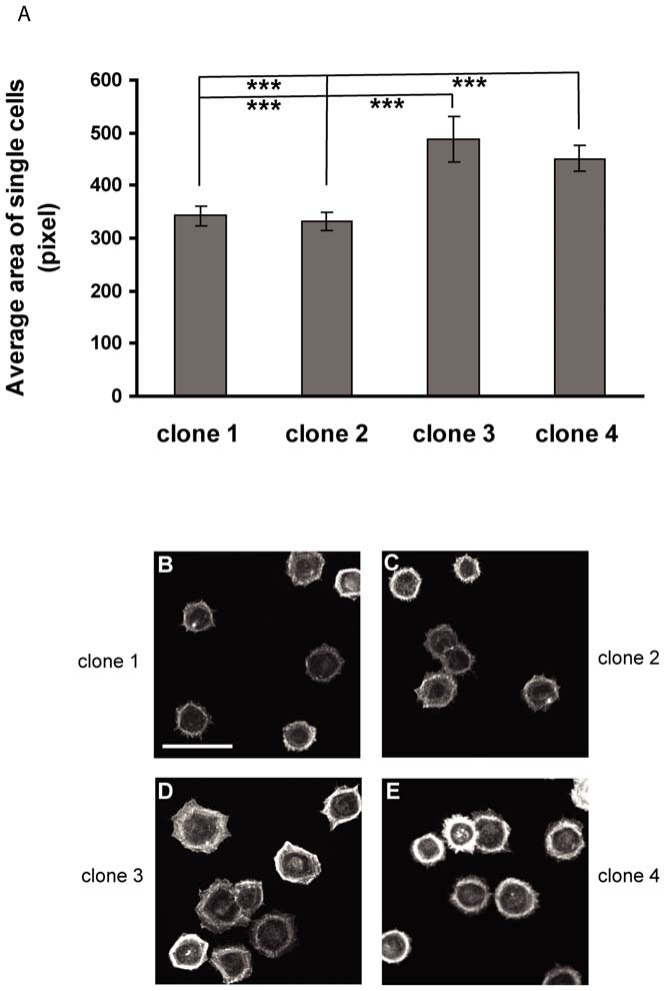
In the absence of SH3PXD2B cell spreading is impaired. (**A–E**) The HeLa clones described in Figure 6 were seeded onto fibronectin-coated coverslips, fixed and permeabilized after 30 minutes and stained with TRITC-Phalloidin. Cell surface area was calculated based on images of over 200 cells using the ImageJ 1.38x software. Average size of the cells for two SH3PXD2B-negative (clones 1 and 2) and two control cell lines (clones 3 and 4) is plotted (**A**). Panels B-E show representative images of poorly spread (**B and C**) and well spread (**D and E**) HeLa cells. Error bars represent the Standard Error of the Mean, SEM. ***: p<0.001, n = 6 Bar represents 20 µm.

The lack of SH3PXD2B expression in many non-adherent cell lines and primary cells (e.g. lymphocytes or unstimulated monocytes) and its association with the actin cytoskeleton suggested that SH3PXD2B may play a role in cell adhesion to extracellular matrices and/or cell spreading. To examine the effect of SH3PXD2B on spreading of transformed cell lines we used the previously described HeLa lines in which SH3PXD2B expression was inhibited by constitutively expressed specific shRNA. SH3PXD2B-negative or control cells were placed on fibronectin-coated coverslips, allowed to attach and spread for various times including 30 minutes, one hour or 12 hours. Cells were visualized by actin filament staining. Cell sizes were quantified by image analysis of at least 200 cells on each image. We found that SH3PXD2B negative clones (clones 1 and 2 in **Figure 7A**) spread poorly on fibronectin compared to clones expressing SH3PXD2B (clones 3 and 4 in **Figure 7A**). A consistent, approximately 25% reduction in the average cell size is apparent even by visual inspection (**Figure 7B–E**).

Together, these experiments demonstrate that SH3PXD2B is one of the key components of the molecular assemblies regulating the development of actin-based membrane structures essential for cellular attachment and spreading.

## Discussion

Development of membrane protrusions such as lamellipodia and membrane ruffles requires the concerted action of several signaling proteins and phospholipids [44], [45]. In this paper we report that SH3PXD2B is an important component of the signaling machinery regulating the formation of these membrane structures. SH3PXD2B is a recently described homolog of FISH/Tks5, a Src substrate required for the organization of Src-induced podosomes [7]. Both proteins are characterized by the presence of an N-terminal PX-domain, followed by four SH3 domains in SH3PXD2B, and five SH3 domains in FISH/Tks5. FISH/Tks5 was described to localize to podosomes of Src-transformed fibroblasts [32]. An active involvement of FISH/Tks5 in the organization of podosomes was also demonstrated by Seals et al. showing that siRNA mediated downregulation of FISH/Tks5 in Src-transformed cells inhibited podosome formation and reduced invasiveness [7]. Podosomal localization [26], [31] of SH3PXD2B and its role in controlling matrix degradation by Src-induced podosomes of murine embryo fibroblasts have also been reported. Data concerning the characteristics of the human orthologue of SH3PXD2B are sporadic, and little is known about the role this novel adaptor protein may play in the organization of other actin-based membrane organelles.

Based on its association with podosomes, it is not unexpected that we found SH3PXD2B highly expressed in multiple tumor-derived cell lines **(** **Figure 1A** **)**. Analysis of the subcellular localization of the protein in unstimulated HeLa and Cos-7 cells revealed an abundant cytoplasmic staining. In the presence of epidermal growth factor SH3PXD2B associated with EGF-induced membrane ruffles and lamellipodia **(** **Figure 2A–F** **)**.

We also pursued to map the structural requirements for the translocation of SH3PXD2B in response to EGF. Based on the presence of the N-terminal PX-domain we hypothesized that a lipid-protein interaction might be the driving force behind SH3PXD2B translocation. PX-domains are lipid-binding modules, present in several proteins involved in signaling and trafficking [41] binding to phosphorylated PtdIns. According to our experiments, the PX-domain of SH3PXD2B binds to all three PtdIns-monophosphates, -diphosphates, and PtdIns(3,4,5)P_3_ detected by using purified recombinant GST-PX-domain in protein-lipid overlay assay experiments. Interestingly, a relatively strong binding of PtdIns(3,5)P_2_ compared to PtdIns(3,4)P_2_ was detected in compliance with the data reported by Hishida et al. [29], and to some extent contradicting the results of the Courtneidge group [26] showing strong binding to PtdIns(3,4)P_2_ but not to PtdIns(3,5)P_2_. Importantly, binding to PI(3,4,5)P3 was also detected which was not detected by either group using the murine protein.

Next we examined the EGF-induced membrane translocation of the full-length protein which carried a deleterious mutation in the PX-domain. Mutation of a conserved residue (R43) located within the PX-domain had no effect on the localization of the full-length protein, suggesting that phospholipid-protein interaction is not the major driving force targeting SH3PXD2B to actin-rich membrane protrusions (**Figure 5D–I**). This finding challenges the generally accepted idea that the PX-domain and its binding affinity to particular phosphatidylinositol phosphates targets PX-domain containing proteins into appropriate membrane compartments. Thus, in the case of SH3PXD2B, protein interactions may play a dominant role in appropriate cellular targeting. In line with this, SH3PXD2B is not the only PX/SH3-domain containing protein where subcellular targeting is under the control of protein interactions. PI3-kinase dependent indirect membrane targeting of p47^phox^ via interaction with moezin was suggested by Zhan et al. [46], [47]. Although we found binding of phosphorylated PtdIns dispensable for the translocation of SH3PXD2B into lamellipodia, lipid binding by the PX-domain still could be responsible for the stabilization of the protein in the above structures. In line with this, upon activation by various chemoattractants, phosphoinositides including PtdIns(3,4,5)P_3_ are enriched in lamellipodia formed at the leading edge of migrating cells [9]. This latter finding also underlines the potential significance of PtdIns(3.4.5)P_3_ binding by the PX-domain.

In our work several observations indicate that the presence of SH3PXD2B in lamellipodia and membrane ruffles and its co-localization with cortactin in these structures is not accidental; rather, SH3PXD2B is requisite for the formation of these structures. Elimination of SH3PXD2B in HeLa cells by RNA interference strongly reduced EGF-induced lamellipodia formation and also inhibited the characteristic membrane localization of cortactin (**Figure 6H–K**). When SH3PXD2B expression in HeLa cells was inhibited, the cells spread less efficiently on fibronectin coated surfaces indicating a substantial role of SH3PXD2B in this process (**Figure 7A–E**).

These observations indicate that the role of SH3PXD2B is not restricted to the regulation of podosome formation. Our experiments revealed protein-protein interactions that might be crucial in SH3PXD2B action. We showed that SH3PXD2B co-localized with cortactin in EGF-induced membrane ruffles and was part of a complex containing cortactin, a well-known Src-substrate with a central role in the organization of the cortical actin cytoskeleton (**Figure 2D–F** **&** **4B**). Cortactin is a scaffold protein, which brings together several proteins at the sites of actin polymerization [20], [48]. The N-terminal part of cortactin contains an N-terminal acidic region that binds to the Arp 2/3 complex and a repeat region with sites for F-actin binding. The C-terminal part contains a proline-rich region with Src-phosphorylation sites and an SH3 domain. This region of cortactin has been reported to bind several proteins, including WASP, dynamin 2 and WIP [20].

Although we cannot exclude that association of SH3PXD2B with cortactin requires other proteins, it is tempting that the SH3-domain of cortactin binds to one of the proline-rich regions on SH3PXD2B. Indeed, in pull-down experiments we localized the region required for the interaction between SH3PXD2B and cortactin to the C-terminal part (amino acids 336–542) of cortactin (**Figure 4C**) and showed that this interaction required the presence of an intact SH3-domain on cortactin (**Figure 4C** **bottom panel**). Importantly, the SH3-domain of cortactin has also been shown to interact with FISH/Tks5 [49]. Surprisingly, association of SH3PXD2B with cortactin did not require tyrosine-phosphorylation of either protein (compare **Figure 4B** **with 4C**). We speculate that, similar to p47^phox^, induction of the functionally active conformation of SH3PXD2B may require phosphorylation of serine residues of SH3PXD2B, cortactin or both. In support of this, EGF-stimulation of tumor cells leads to phosphorylation of cortactin at serine residues 405 and 418 inducing a characteristic shift in cortactin electrophoretic mobility from 80 kDa to 85 kDa [50], [51]. Phosphorylation of these serine residues by ERK1/2 has recently been shown to be requisite for efficient tumor cell motility, adhesion and lamellipodia persistence [52]. It is tempting to speculate that the phosphorylation by ERKs or other kinases on appropriate serine residues may be required for the translocation of SH3PXD2B to the membrane. By the interaction of SH3PXD2B with cortactin, a complex interface of five SH3 domains, two proline rich regions, at least seven tyrosine and multiple serine residues may be formed representing a binding surface with exceptional complexity. Association of SH3PXD2B with cortactin-containing complexes brings SH3PXD2B to close proximity with cortactin-associated proteins, many of which may prove to be regulated by SH3PXD2B.

Although detailed description of the molecular scaffold of SH3PXD2B and cortactin is yet ahead of us, we identified SH3PXD2B as a novel interacting partner of Src and cortactin. Via modulating the cellular localization or the activity of cortactin SH3PXD2B may be a key regulator of cellular attachment and migration during the course of development, in the process of the normal immune response, or in migration and attachment of transformed cells. We think that our findings broaden our view on the multiple developmental defects observed in the absence of SH3PXD2B both in humans and mice [30], [31].

## Materials and Methods

### Cells and antibodies

Human umbilical vein endothelial cells (HUVECs) were purchased from Cascade Biologics. Human monocytes were obtained from leukocyte-enriched buffy coats obtained from healthy volunteer blood donors drawn at the Regional Blood Center of Hungarian National Blood Transfusion Service (Debrecen, Hungary) in accordance with the written approval of the Director of the National Blood Transfusion Service and the Regional and Institutional Ethics Committee of the University of Debrecen, Medical and Health Science Center (Debrecen, Hungary). Written informed consent was obtained from the donors prior to blood donation, and their data were processed and stored according to the principles expressed in the Declaration of Helsinki. Human monocytes were isolated as described previously [53]. Macrophages were differentiated in the presence of 50 ng/ml macrophage colony-stimulating factor (M-CSF, Peprotech). The Rat-1 LA29ts cells were a generous gift of Margaret Frame [36]. The A2058 melanoma cell line was generously shared with us by Margit Balazs [54]. All other cell lines including HeLa, 293T, COS7, Jurkat, REH, THP1 and TF1 cells, were obtained from American Type Culture Collection (ATCC) and were maintained according to the distributor's instructions. Media for the maintenance of TF1 cells were supplemented with 5 ng/ml granulocyte-macrophage colony-stimulating factor (GM-CSF, Peprotech). The following antibodies were used in this study: anti-actin (SIGMA), antiphospho-tyrosine (4G10, PT66, Sigma) and anti-cortactin (Upstate), anti-V5 (AbD Serotec), anti-cSrc (SantaCruz).

### DNA constructs

To create V5-tagged SH3PXD2B construct the full-length SH3PXD2B coding sequence was cloned into a pcDNA3.1/TOPO-V5-His plasmid (Invitrogen). To create fluorescent protein tagged SH3PXD2B construct the full-length SH3PXD2B coding sequence was cloned into the pEGFP-N1 vector (Clontech). An EGFP-tagged SH3PXD2B PX-domain was created by cloning SH3PXD2B cDNA encoding the first 130 amino acids of the protein into pEGFP-N1 vector. For the production of a SH3PXD2B PX-domain-GST fusion protein, the same cDNA was cloned into a pGEX4T1 plasmid. The R43Q, single amino acid change was introduced into the full-length SH3PXD2B-V5 construct using the Stratagene's Quikchange site directed mutagenesis kit following the manufacturer's instructions. Constructs were verified by sequencing. To generate constructs encoding various segments of GST-cortactin fusion proteins the full-length cortactin (mouse cDNA) was amplified by PCR and subcloned into *BamH1/EcoRI* sites of pGEX-4T-1 vector (GE Healthcare). cDNAs corresponding to the N-terminal part (amino acids 1–334) and C-terminal part (amino acids 336–542) of cortactin were also amplified by PCR and subcloned into *EcoRI/XhoI* sites of pGEX-4T-1 plasmid. The Src construct was a generous gift of Julian Downward (Cancer Research UK, London) and was used earlier [55].

### Production and affinity purification of SH3PXD2B-specific antibody

Polyclonal SH3PXD2B-specific antibody was purified from rabbit serum following intra-cutaneous injections of GST-SH3PXD2B (amino acids 23–430) fusion protein into rabbits. SH3PXD2B-specific antibodies were purified from the rabbit sera in two steps. First, GST-specific antibodies were depleted from the immune serum using recombinant GST protein coupled to glutathione sepharose beads followed by an affinity purification step using Affigel 10 beads (BioRad Laboratories) loaded with the antigen.

### Transient transfection, immunoprecipitation, western blotting

293T cells were transfected by the standard calcium-phosphate method. Cells were seeded at the density of 3x10^6^ cells on 100 mm plates. Next day, cells were transfected with 20 µg SH3PXD2B-V5 and 5 µg c-Src either alone or in combination. To equalize the amount of DNA each transfection reaction was supplemented with the appropriate empty vectors. 48 hours post-transfection cells were lysed in RIPA buffer (1% Triton X-100, 0.5% Deoxy-cholate and 0.1% SDS) containing phosphatase inhibitor mix (Sigma), protease inhibitors: Aprotinin, Leupeptin, Pepstatin, Bestatin (20 ug/ml each) and 1 mM PMSF. Lysates cleared from detergent insoluble material by centrifugation at 15,000× g for 10 min at 4°C, were incubated overnight at 4 ^0^C with 4 µg anti-V5 antibody (AbD, Serotec) and 30 µl of ImmunoPure Immobilized Protein G beads (Pierce) or with glutathion-agarose beads (GST pulldown experiments). Immunoprecipitates were washed four times with ice-cold 50 mM HEPES buffer, pH 7.4, containing 150 mM NaCl, 0.2% Triton X-100. Precipitated complexes were subjected to SDS-PAGE and transferred to polyvinylidine difluoride membranes (Immobilon-P, Millipore). Membranes were blocked with 3% BSA (Sigma), incubated with the indicated primary antibodies and horseradish peroxidase-conjugated secondary antibodies (Amersham), and developed using enhanced chemiluminescence (ECL West Pico, Pierce). For precipitation with GST fusion proteins 5 µg of the specified GST-cortactin proteins were used as non-covalently bound adducts to glutathione-agarose beads. Separation of bound proteins and immunoblotting were performed as described previously [56].

### Downregulation of SH3PXD2B expression in HeLa cells by RNA interference

For transient transfection experiments SH3PXD2B-specific Stealth® siRNAs and “minimally” changed, control siRNAs were obtained from Invitrogen. The target sequences on SH3PXD2B mRNA were the followings: GCCTGATACCAATTGATGAATACTG (Si2-SH3PXD2B), GCTGGTGGTACATTCAGATTGAAGA (Si3-SH3PXD2B). The control sequences were the followings: GCCGGATACCAATTGATTAACATTG (Si2controlSH3PXD2B) GCTAGTGGTACGTTCAGATTGGAAA (Si3controlSH3PXD2B). Nucleotides altered to obtain the control oligonucleotides are underlined. Si2-SH3PXD2B targets exon 4 encoding part of the PX-domain, while Si3-SH3PXD2B targets exon 13 located within the region encoding the third SH3-domain of SH3PXD2B. siRNAs were transfected into Hela cells at 100 nM using the RNAiMax transfection reagent (Invitrogen). Cells were routinely analyzed 48 hours after transfection. To create HeLa cell lines stably expressing SH3PXD2B-specific siRNAs we used the siSTRIKE system from Promega. The double-stranded oligonucleotides representing the si3-SH3PXD2B or si3control-SH3PXD2B target sequences together with sequences required to form the double-stranded loops of the shRNA were cloned downstream of the U6 promoter region of the siSTRIKE-Puro vector as suggested by the manufacturer. 10^6^ cells were transfected with siSTRIKE plasmid expressing SH3PXD2B-specific siRNA (or mutant construct as a control) using Fugene 6 transfection reagent (Roche). 72 hours past transfection stable transfectants were selected in the presence of 1 µg/ml puromycin for 1 week. Next, single cell clones were isolated by limiting dilution in the presence of 1 µg/ml puromycin. SH3PXD2B expression was analyzed by PAGE and western blotting using the affinity purified antibody. Immunoblots were developed with the affinity-purified SH3PXD2B-specific antibody described above. β-actin was used as a loading control. We have isolated 20 puromycin-resistant HeLa clones which were further studied.

### Immunofluorescent labeling and confocal laser microscopy

Cells grown on coverslips were fixed in 4% paraformaldehyde in PBS then washed 5 times in PBS and incubated for 10 minutes in PBS containing 100 mM glycine. Coverslips were washed 2 times in PBS and permeabilized in PBS containing 1% BSA and 0.1% Triton X-100 for 20 minutes at room temperature. After 1 hour blocking in PBS containing 3% BSA cells were incubated with the primary antibody in PBS plus 2% BSA, washed thoroughly 6 times in PBS and incubated with the secondary antibody for 1 hour and finally washed 6 times in PBS again. Coverslips were mounted using Mowiol 4–88 antifade reagent (prepared from polyvinyl alcohol 4–88, glycerol, H_2_O and Tris pH 8.5).

Confocal images were collected on an LSM510 laser scanning confocal unit (Carl Zeiss) with a 63×1.4 numerical aperture plan Apochromat and a 40×1.3 numerical aperture plan Neofluar objective (Carl Zeiss). Excitation was with 25 mW argon laser emitting 488 nm, and a 1.0 mW helium/neon laser emitting at 543 nm. Emissions were collected using a 500–530 nm band pass filter to collect A488 and GFP and a 560 nm long pass filter to collect A568 and RFP emission. Usually images from optical slices of 1–2 µm thickness were acquired. Crosstalk between fluorophores was negligible.

### Gelatin degradation assay

Coverslips were coated with 1 mg/ml gelatin conjugated with Oregon Green488 (Molecular Probes, G13186) dissolved in 2% sucrose at 4°C for 20 minutes in the presence of glutaraldehyde. After washing three-times in PBS coverslips were incubated with 5 mg/ml NaBH4 (dissolved in PBS) at RT for 3minutes, washed again as above and sterilized and dried using 70% ethanol. Rat-1 LA29 fibroblast cells carrying a temperature sensitive mutant form of Src were grown at the nonpermissive temperature for 24 hours then seeded onto coverslips coated with the conjugated gelatin and incubated for 9 hrs at either the nonpermissive or at the permissive temrerature. Coverslips were prepared for immunfuorescence as described above.

### Lipid binding assay

PIP strips were obtained from Invitrogen. Protein-lipid overlay assay experiments were performed using SH3PXD2B PX(1–130)-GST and GST fusion proteins following the manufacturer's protocol. Layout of the PIP strip was as follows: **1**. Lysophosphatidic acid **2**.Lyso-phosphatidylcholine **3**. Phosphatidylinositol (PtdIns) **4**. PtdIns(3)P **5**. PtdIns(4)P **6**. PtdIns(5)P **7**. Phosphatidylethanolamine **8**. Phosphatidyl-choline (PC) **9**. Sphingosine 1-phosphate **10**. PtdIns (3,4)P2 **11**. PtdIns(3,5)P2 **12**. PtdIns(4,5)P2 **13**. PtdIns(3,4,5)P3 **14**. Phosphatidic acid **15**. Phosphatidylserine **16**. Blank.

### Liposome binding assay

Two lipids, PC and PtdIns(3)P were dissolved in chloroform at the concentration of 10 µg/µl and dried under nitrogen atmosphere. To prepare liposomes, in each experiment 100 µg total lipid was rehydrated at room temperature in 50 µl 10 mM HEPES, 140 mM NaCl, pH 7.4, followed by vortexing until the mixture was homogeneously opalescent. The GST-fusion proteins were centrifuged at 80000 g for 20 min to remove all aggregates. One microliter of supernatant (containing approximately 1 µg protein) was added to 50 µL of liposome solution. Binding was performed at 37°C for 30 min on a laboratory rocker. The vesicles were centrifuged at 80000 g for 20 min. Pellet and supernatant were separated, boiled and then subjected to SDS-PAGE. The protein was detected by western-blotting with a GST-specific antibody. Densitometry on scanned Images was done using the ImageJ 1.38x software.

### Cell spreading on fibronectin

Glass coverslips were coated with fibronectin (20 µg/ml) at 4°C, overnight and washed twice with PBS to remove any unbound fibronectin. To block remaining binding sites, coverslips were then incubated with 5% BSA. HeLa cells were plated onto 12 well plates containing the fibronectin-coated coverslips and fixed by 4% paraformaldehyde at various time points after plating. Fixed cells were permeabilized by 0.2% Triton X-100 in PBS for 5 min followed by phalloidin staining (0.1 µg/ml TRITC-phalloidin in PBS for 20 min). TRITC-phalloidin stained cells were photographed using a Nikon Eclipse E400 fluorescent microscope. At least 200 cells from each slide were analyzed using the ImageJ 1.38x software (NIH, USA).

## Supporting Information

Figure S1 **Src activation induces the development of functionally active podosomes in LA29ts cells**. Rat-1 LA29 fibroblast cells carrying a temperature sensitive mutant form of Src were cultured at the nonpermissive temperature (39.5°C) for 24 hours. Next, cells were seeded onto coverslips coated with OregonGreen488-labeled gelatin and incubated for 9 hrs at the nonpermissive (A–C) or at the permissive (D–F) temperature. Cortactin was visualized as described in the legend for Figure 2. White arrows point to areas where podosome structures still overlap with degrading gelatin (orange color). Dark dot-shaped areas represent previous attachment sites of podosomes where gelatin had been degraded and podosomes detached as cells moved on. Bar represents 20 µm.
(TIF)
