# Supplementary figures and images for: The Homolog of the Five SH3-Domain Protein (HOFI/SH3PXD2B) Regulates Lamellipodia Formation and Cell Spreading
Source: PLoS One. 2011 Aug 23;6(8):e23653. doi: 10.1371/journal.pone.0023653 (PMC3160312; doi:10.1371/journal.pone.0023653)

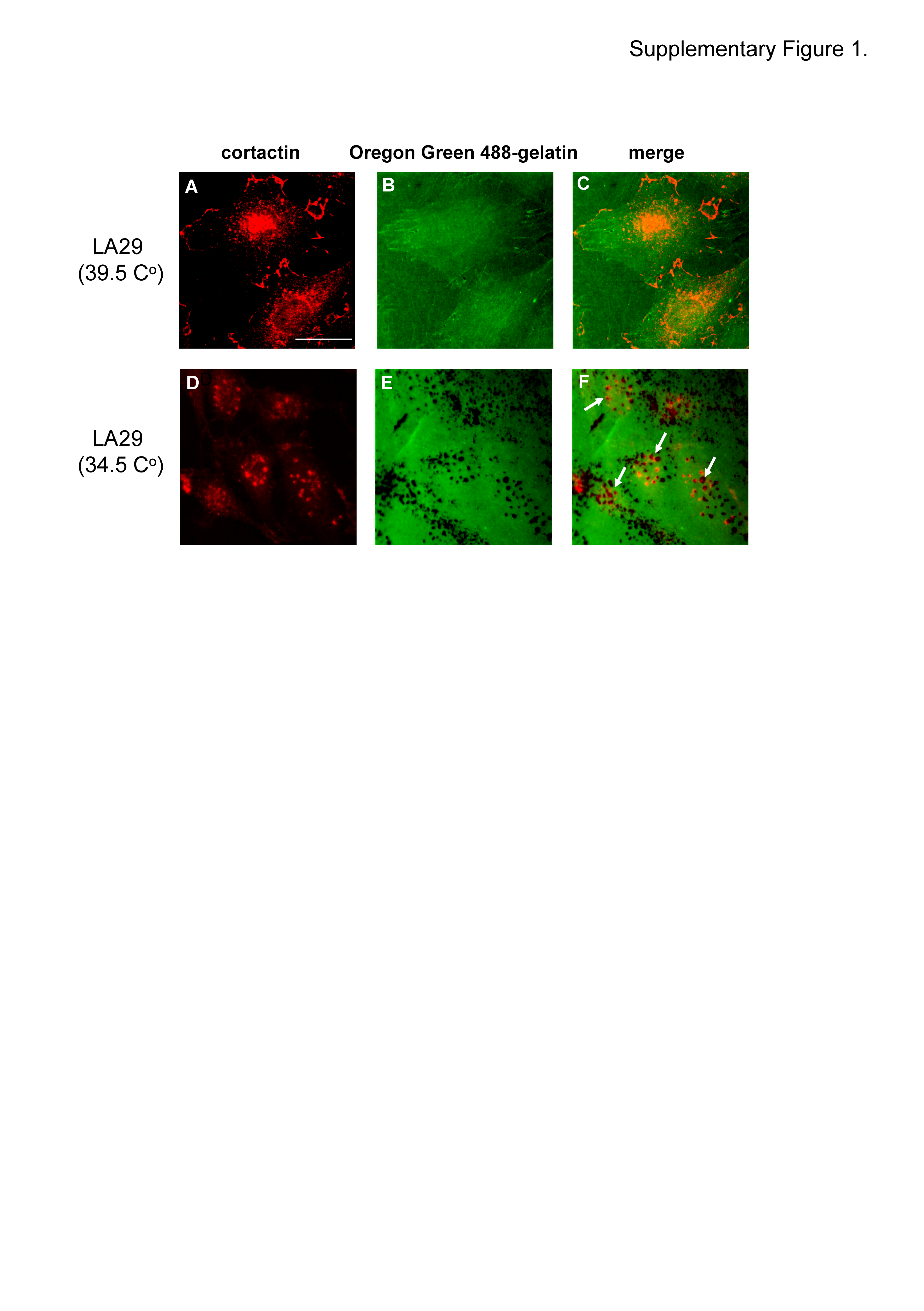

Supplement: Figure S1 — Src activation induces the development of functionally active podosomes in LA29ts cells. Rat-1 LA29 fibroblast cells carrying a temperature sensitive mutant form of Src were cultured at the nonpermissive temperature (39.5°C) for 24 hours. Next, cells were seeded onto coverslips coated with OregonGreen488-labeled gelatin and incubated for 9 hrs at the nonpermissive (A–C) or at the permissive (D–F) temperature. Cortactin was visualized as described in the legend for Figure 2. White arrows point to areas where podosome structures still overlap with degrading gelatin (orange color). Dark dot-shaped areas represent previous attachment sites of podosomes where gelatin had been degraded and podosomes detached as cells moved on. Bar represents 20 µm. (TIF) [file pone.0023653.s001.tif]
